# Measurement of Restitution and Friction Coefficients for Granular Particles and Discrete Element Simulation for the Tests of Glass Beads

**DOI:** 10.3390/ma12193170

**Published:** 2019-09-27

**Authors:** Hongxiang Tang, Rui Song, Yan Dong, Xiaoyu Song

**Affiliations:** 1 State Key Laboratory of Coastal and Offshore Engineering, Dalian University of Technology, Dalian 116023, China; songrui_dlut@163.com; 2 Anhui Transport Consulting & Design Institute Co., LTD., 180 Xiangzhang Avenue, Hefei 230000, China; doongyan@163.com; 3 Engineering School of Sustainable Infrastructure and Environment, University of Florida, 365 Weil Hall, Gainesville, FL 32611, USA

**Keywords:** restitution coefficient, friction coefficient, slant plate flat throw test, glass bead, discrete element, triaxial test, plane strain test

## Abstract

A slant plate flat throw test system for measuring the restitution coefficient of granular materials and a sliding friction test instrument for measuring the friction coefficient between discrete particles and continuum boundary surface materials are developed. The restitution coefficients of the glass bead particles, the glass beads relative to the glass plate, the composite of glass plate and the rubber membrane and the friction coefficients between the glass beads and the rubber film and the filter paper are measured by the designed methods. Based on the measured restitution coefficient and friction coefficient, the discrete element numerical simulation is carried out for triaxial test and plane strain test. Through comparing the experimental results and the discrete element numerical simulation results, the feasibility and rationality of the designed measurement methods and the discrete element numerical simulation are verified. The measuring methods developed in this paper can be further applied to the tests of other fine particles.

## 1. Introduction

In the process of discrete element numerical simulation, the contact relationship between particles and particles and the contact relationship between particles and boundary surfaces are often involved [1]. When calculating the contact relation, the parameters of discrete element particles need to be input first, which involves the calibration of contact parameters. The parameters of particles themselves can be obtained by referring to data or conventional tests. Here, the contact parameters between particles and contact surface, namely the restitution coefficient and friction coefficient, are mainly studied. For the low-speed collision contact problem of a spherical near-rigid object, the restitution coefficient is the material correlation quantity, which indicates the ratio of the normal relative velocity before the collision of the contact point to the normal relative velocity after the collision [2] and in the discrete element simulation process, will affect the value of normal damping and time step. In the process of discrete element numerical simulation, the value of the friction coefficient will have a great impact on the strain localization of the sample [3] and the mechanical properties of particle materials [4]. Therefore, how to obtain the restitution coefficient and friction coefficient between particles and contact surface is very important.

Malla et al. measured the friction coefficient between the particle and the cylinder wall for a laterally confined granular column [5]. Han et al. investigated the friction coefficient between elliptical particles and inclined plate by measuring the inclination angle of particles on inclined plates [6]. Liu measured the rolling friction coefficient and static friction coefficient between iron cylinder and glass by a simple experimental device [7]. Hu et al. studied the influencing factors of sliding friction between rods and particulate matter by dragging upward the rods inserted into particulate matter [8]. Chang et al. studied the friction behavior of a mechanical surface sliding on hard particles [9]. Bek et al. presented an instrument for measuring the friction of granular materials and the effectiveness of the instrument is verified by four kinds of granular materials with different particle sizes and materials [10].

Sun et al. studied the influence factors of particle friction by cutting spherical particles to change the shape of particles and analyzed the rolling-sliding friction transformation mechanism between particles and plates [11]. Orlando studied the influence of shear rate, particle size and particle density on friction coefficient by ring shear test [12]. Nasuno et al. studied the effects of velocity, modulus of elasticity and particle diameter on the friction of slider on the surface of granular layer [13].

For the contact problem of spherical near-rigid object in low-speed impact, the restitution coefficient is the material correlation, which represents the ratio of normal relative velocity before and after particle collision to normal relative velocity after collision [2]. Much work has been done on the research and application of restitution coefficient. In the measurement method, the free-falling body impact test was used to study and discuss the factors affecting the restitution coefficient of particle collision at low speed, including particle mass, falling height [14], characteristics of collision material, thickness [15], velocity of particle collision instantaneous relative to collision material, curvature of contact surface between two collision materials and collision history [16]. The restitution coefficient was also measured by falling stone takeoff method through sound wave [17,18]. In the study of restitution coefficient of material with a certain thickness of liquid layer on particle and surface, the influence of thickness of liquid layer on restitution coefficient was discussed and the influence of Stokes number on restitution coefficient was explained [19,20,21]. In the measurement of restitution coefficient of non-spherical materials, the effects of random angular direction, eccentricity and degree of freedom of rotation on restitution coefficient [22] and the effects of different exit angles and angular velocities on restitution coefficient of polyethylene cylinder particles [23], were discussed. In terms of particle form, the restitution coefficients of unbonded particles, dry particles and wet particles have been studied and a calculation model has been proposed based on the experimental results [24]. Coke particle [25], snow particle [26], gas particle [27] are also affected by many factors in the measurement of restitution coefficient. In order to deal with natural disasters, the restitution coefficient of rolling stones on slopes [28,29,30] was measured to prevent the disasters caused by landslides. The restitution coefficient of ship collision was analyzed and used to analyze the disaster caused by collision between ship and pier in water [31].

This paper mainly developed a slant plate flat throw test system for measuring the restitution coefficient of granular materials and a sliding friction test instrument for measuring the friction coefficient between discrete particles and continuum boundary surface materials. The purpose of particle material parameter measurement is to be used for numerical simulation under relevant working conditions. As the preliminary stage of the study, this paper mainly tests the parameters of the glass bead and simulates the triaxial test and plane strain test in laboratory with proposed discrete element method (DEM). By applying the measured parameters to the discrete element numerical simulation of triaxial and plane strain model test, the feasibility and rationality of the designed measurement methods and the discrete element numerical simulation are verified.

## 2. Measurement Methods of the Restitution Coefficient and Friction Coefficient for Particles

### 2.1. Properties of the Test Materials

The discrete element model involves some input of initial parameters. In order to make the numerical simulation more accurate, the selected parameters should be as consistent as possible with the parameters of actual particles. The ordinary parameters used in DEM will be given in the numerical analysis part. The non-ordinary parameters such as the restitution coefficients between the glass bead particles, between the glass bead and the porous stone and the rubber membrane, are measured by a slant plate flat throw test system developed in this paper; the friction coefficients between the glass beads and the rubber membrane and the filter paper are measured by a feasibly designed method.

**Figure 1:**
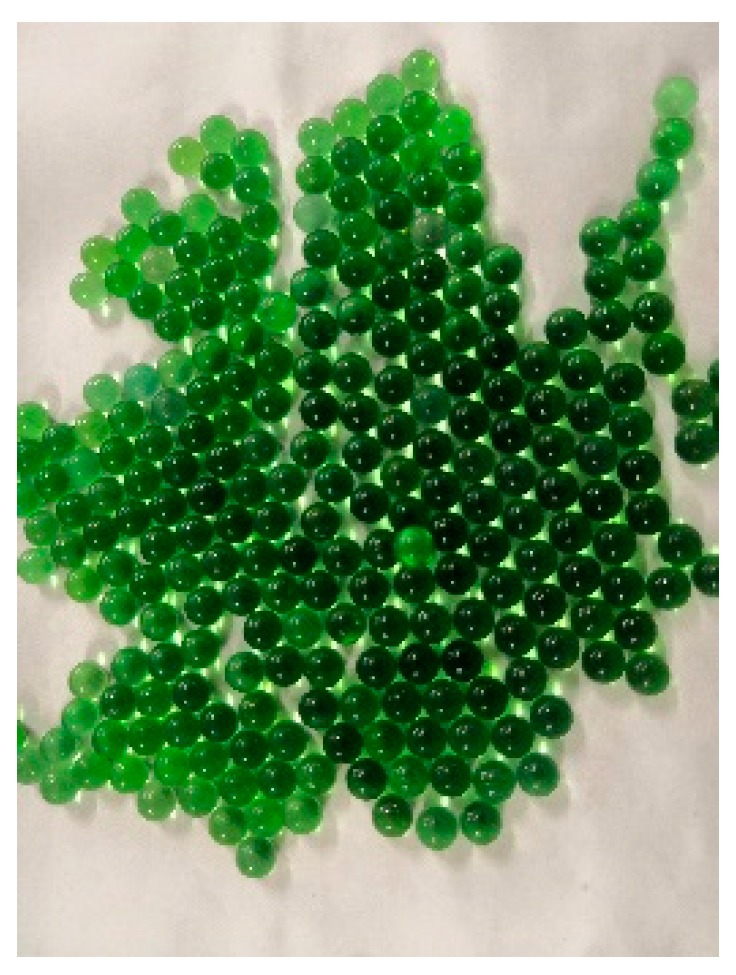
Glass beads used in the test.

**Figure 2:**
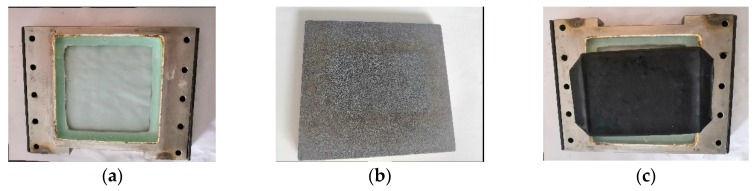
Test materials: (**a**) glass plate, (**b**) porous stone and (**c**) glass plate with rubber membrane.

The glass beads used in the test are shown in Figure 1 and the physical properties of the glass beads are shown in Table 1. They are taken from the manufacturer (Yiyi Handicraft factory, Yiwu, Zhejiang, China, https://m.tb.cn/h.eNque2J?sm=78c443). During the triaxial test and the plane strain test, the materials directly contacting the glass beads mainly include a plane strain rubber film, filter paper and indirect contact materials are water-permeable stones and glass plates. The test materials are shown in Figure 2 and the thickness of the material is shown in Table 2.

### 2.2. Measurement of the Restitution Coefficient between Glass Beads and Other Materials

**Figure 3:**
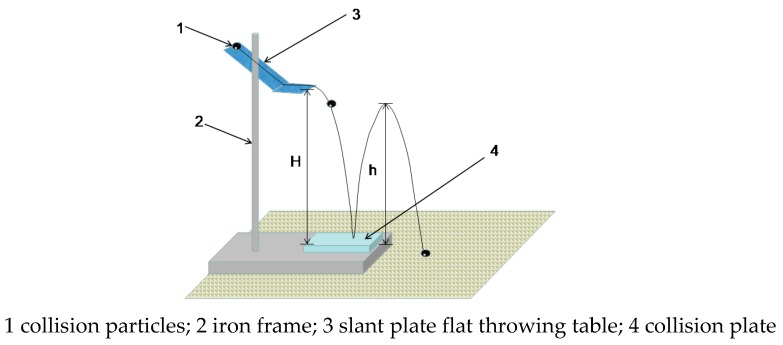
Schematic diagram of slant plate flat throw test system.

The restitution coefficient is generally expressed by *COR* (coefficient of restitution) and its measurement principle is shown in the slant plate flat throw test system shown in Figure 3. The use of a flat throwing motion, rather than a free fall, ensures the stability of the vertical velocity when in contact with the impact plate.

The testing equipment involved in the test process includes: camera, iron frame, flat throwing table (consisting of a transparent plastic plate with a certain inclination angle and a horizontal transparent plastic plate), scale paper, graduated scale, electronic digital caliper, the water pressure sensors, tweezers, level bubble (to ensure that the particles can be thrown horizontally each time) and materials including glass plate collision, waterproof rubber membrane attached to porous stone, glass and so forth. It can be concluded that the restitution coefficient is equal to the arithmetic square root of the ratio of the height of the parabolic vertex from the collision plate to the height of the plane throw from the collision plate, as illustrated in Formula (1).

(1) $$COR=\sqrt{\frac{h}{H}}$$

where: *h* represents the height of the parabola apex from the collision plate and *H* represents the distance from the particle throwing point to the collision plate.

The two-dimensional motion trajectory analysis can be used to measure the restitution coefficients between the glass beads and various materials, as shown in Table 3. The restitution coefficients will be used for the DEM numerical simulation and to calculate the viscous damping coefficients of normal force and sliding force, as shown in Formulas (7) and (8) thereafter.

### 2.3. Friction Coefficient Measurement

**Figure 4:**
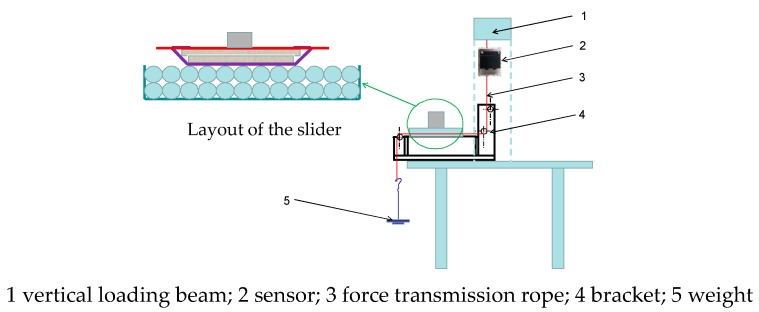
Schematic diagram of the test of sliding friction coefficient.

The diagram of the integral loading device for measuring the friction coefficient is shown in Figure 4. During the test, the horizontal movement of the slider is controlled by the up and down movement of the added beam. When measuring the friction coefficient between the particles and the sliding block, according to the weight of the sliding block and the friction force measured by the weight of the corresponding sliding block (Sliding block friction = tension on the rope when there is a sliding block—tension on the rope when there is no sliding block, the pressure-friction curve is drawn and the friction coefficient is obtained. The pressure-friction curve should go through the origin. According to the friction-sliding speed curve of the slider, the friction coefficient curve of the particles in quasi-static state is obtained and the friction coefficient at the quasi-static state is determined.

**Figure 5:**
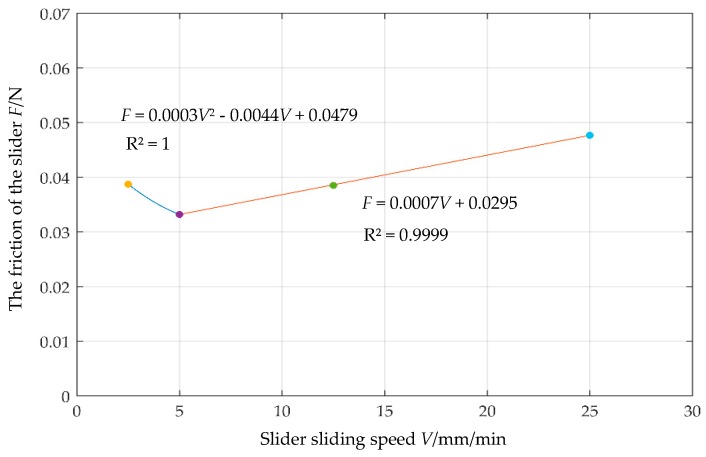
Friction-sliding speed curve of the slider.

During the test, the relative movement speed between the particles and the boundary is very slow. Therefore, before studying the friction coefficient between the glass beads and the boundary, the influence of the sliding velocity on the sliding friction coefficient is first investigated. Four sliding speeds were selected, respectively 25 mm/min, 12.5 mm/min, 5 mm/min and 2.5 mm/min. The force measurement results of the slider and the rope are shown in Figure 5. As the sliding speed *V* increases, the measured frictional force *F* increases slightly but the increase is small. The curve can be divided into two sections with two different fitting formulas, one of which is the linear one and another is nonlinear at low speeds and can be fitted as

(2) $$\begin{array}{l} F=0.0003V^{2}-0.0044V+0.0479\;0\leq V\leq 5\\ F=0.0007V+0.0295\;V>5 \end{array}$$

Among them, the intercept of 0.0479 N can be considered as the frictional force of the particles under quasi-static conditions. It can be seen that at a small rate, the sliding speed has little effect on the friction of the slider between the particles and the boundary surface.

**Figure 6:**
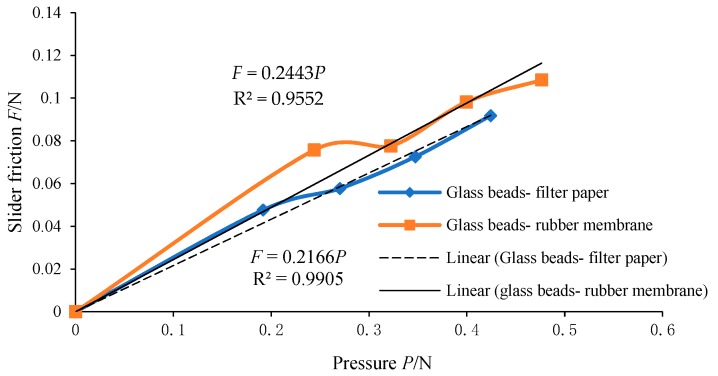
Sliding friction - pressure curve.

During the test, filter paper and rubber membrane are directly in contact with glass beads. The test materials used were the plane strain test rubber membrane and the filter paper for the routine test. Among them, the side in contact with the particles was used in the test of the rubber membrane. For the measurement of the friction coefficient between the contact material and particles, the tension - pressure curve is obtained by changing the weight of the sliding block and the friction coefficient is obtained by curve fitting. The final measured curve is shown in Figure 6.

**Figure 7:**
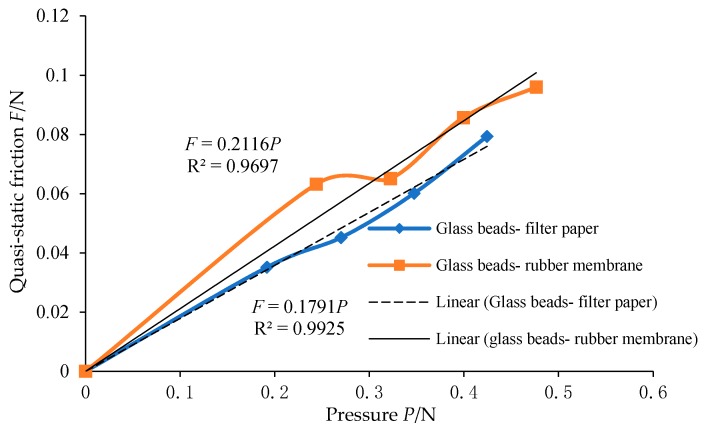
Quasi-static friction-pressure curve.

It can be seen from Figure 6 that, with the increase of pressure *P*, the friction force *F* of the sliding block is approximately linear. The slope of the fitting straight line is the friction coefficient of the sliding block when its sliding speed is 25 mm/min. According to formula (2), the friction of the sliding block in quasi-static state can be calculated and the curve shown in Figure 7 can be drawn. It can be seen from Figure 7 that the sliding velocity has an influence on the friction coefficient between particles and the boundary surface at a small rate. The friction coefficient between glass beads and different materials is shown in Table 4.

## 3. Discrete Element Simulation for Triaxial Test

### 3.1. Discrete Element Model

**Figure 8:**
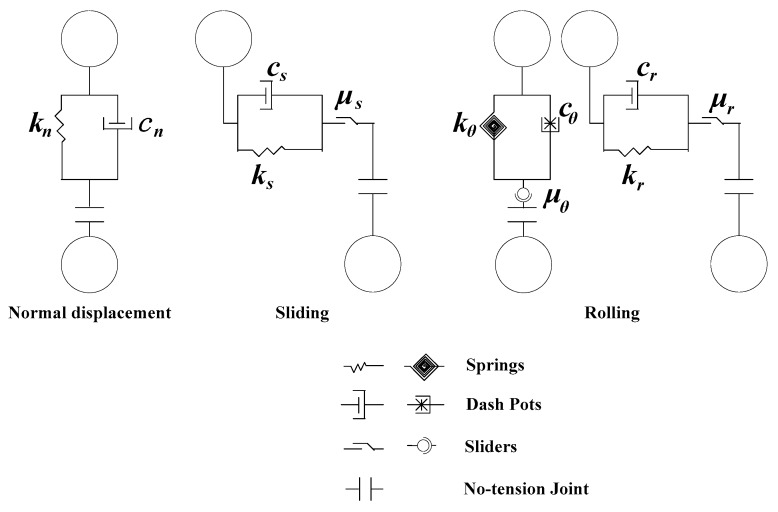
Contact model incorporating both rolling resistances and sliding frictional force (according to Tang et al., 2016).

The discrete element method (DEM) has been widely used to investigate the failure micro-mechanism of granular particles because of the ability to obtain the microscopic information at the particle level [32,33]. DEM allows for a contact model between two particles, viz. force-displacement law, for calculating the contact force. The physical properties and relative motion mechanism of particles are reflected by contact models between particles. Using different contact models, different macroscopic behaviors can be achieved with DEM. A repulsive normal force between two particles in contact arises due to the stiffness of the particles. A contact model for the calculation of tangential forces between two particles in contact, which incorporates both rolling resistances and the sliding resistances, is used in this study [34,35]. This model is shown in Figure 8.

The normal contact force, tangential contact force and contact torque between two particles are calculated as:

(3) $$\{\begin{array}{c} \begin{array}{c} F_{n}=k_{n}U_{n}+c_{n}\frac{dU_{n}}{dt}\\ F_{t}=F_{s}+F_{r}=\min(k_{s}U_{s}+c_{s}\frac{dU_{s}}{dt},\mu_{s}\left|F_{n}\right|)+\min(k_{r}U_{r}+c_{r}\frac{dU_{r}}{dt},\mu_{r}\left|F_{n}\right|) \end{array}\\ m_{r}=-\min(k_{\theta }\theta_{r}+c_{\theta }\frac{d\theta_{r}}{dt},\mu_{\theta }\left|F_{n}\right|) \end{array}$$

where $F_{n}$ is the normal contact force, $F_{t}$ is the tangential contact force, $F_{s}$ is the tangential sliding friction force, $F_{r}$ is the tangential rolling friction force, $m_{r}$ is the rolling friction resistance moment, $k_{n}$ is the stiffness coefficient of the normal force, $k_{s}$ is the stiffness coefficient of the sliding force, $k_{r}$ is the stiffness coefficient of the rolling force, $k_{\theta }$ is the stiffness coefficient of the rolling moment, $U_{n}$ is the normal displacement, $U_{s}$ is the tangential sliding displacement, $U_{r}$ is the tangential rolling displacement, $\theta_{r}$ is the rolling angle, $c_{n}$ is the viscous damping coefficient of the normal force, $c_{s}$ is the viscous damping coefficient of the sliding force, *c*_r_ is the viscosity damping coefficient of the rolling force, $c_{\theta }$ is the viscosity damping coefficient of the rolling moment, $\mu_{s}$ is the sliding friction force coefficient, $\mu_{r}$ is the rolling friction force coefficient and $\mu_{\theta }$ is the rolling friction moment coefficient.

### 3.2. The Parameters for Discrete Element Model

The input of some initial parameters is involved in the discrete element model. In order to make the numerical simulation more accurate, the selected parameters should be as close as possible to the actual particle parameters. These parameters are measured by experiments. Young’s modulus and Poisson’s ratio of granular materials such as glass beads can be obtained by inquiring relevant data, particle radius can be measured by vernier caliper, density of glass particles can be measured by specific gravity test. The friction coefficient between glass beads can be obtained by direct shear test. The restitution coefficient between glass bead particles and rubber film (in this case, glass plate attached to rubber film) and filter paper (samples are indirectly contacted by filter paper and porous stone) can be obtained from the restitution coefficient test designed in this paper. When measuring the friction coefficient between particles and materials, the influence of filter paper is taken into account. When measuring the restitution coefficient between particles and materials, the influence of porous stone is taken into account. When measuring the friction and restitution coefficients between particles and materials, the influence of filter paper should be taken into account. The friction coefficient between glass bead particles and rubber film (in this case, glass plate is attached to rubber film) and filter paper (the sample is indirectly contacted by filter paper and porous stone) can be obtained from the friction coefficient test designed in this paper. The parameters obtained are shown in Table 5, which will be used in DEM analysis for triaxial test and plane strain test.

Then, the other parameters for discrete element model are determined as:

The stiffness coefficient of normal force

(4) $$k_{n}=\frac{r_{A}r_{B}}{r_{A}+r_{B}}\times \pi E$$

The stiffness coefficient of sliding force

(5) $$k_{s}=RNS\times \frac{r_{A}r_{B}}{r_{A}+r_{B}}\times \pi E$$

The stiffness coefficient of rolling moment

(6) $$k_{\theta }={\left(\frac{2r_{A}\times r_{B}}{r_{A}+r_{B}}\right)}^{2}\times k_{s}$$

The viscous damping coefficient of normal force

(7) $$c_{n}=2\sqrt{k_{n}\times m}\times \frac{-COR}{\sqrt{\pi^{2}+{\left(\mathrm{lg}COR\right)}^{2}}}$$

The viscous damping coefficient of sliding force

(8) $$c_{s}=RZS\times 2\sqrt{k_{n}\times m}\times \frac{-COR}{\sqrt{\pi^{2}+{\left(\mathrm{lg}COR\right)}^{2}}}$$

The viscosity damping coefficient of rolling moment

(9) $$c_{\theta }=\frac{-0.2}{1.15344\times 1.5\times {\left(k_{n}/m\right)}^{0.4}}\times F_{N}$$

The rolling friction moment coefficient

(10) $$\mu_{\theta }=ARF\times \sqrt{r_{B}{}^{2}-\frac{{\left(D_{S}{}^{2}+r_{B}{}^{2}-r_{A}{}^{2}\right)}^{2}}{4D_{S}{}^{2}}}$$

where *E* is Young’s modulus, *r*_A_ and *r*_B_ are radius for two contacting particles, *D*_S_ is the distance between the centers of two particles, *m* is the mass of particle.

In the previous section, the measurement of the restitution coefficient and friction coefficient between the glass beads and the boundary was introduced. In order to verify the rationality of the discrete element parameters selected, this part carried out the discrete element numerical simulation analysis of the glass bead triaxial test.

**Figure 9:**
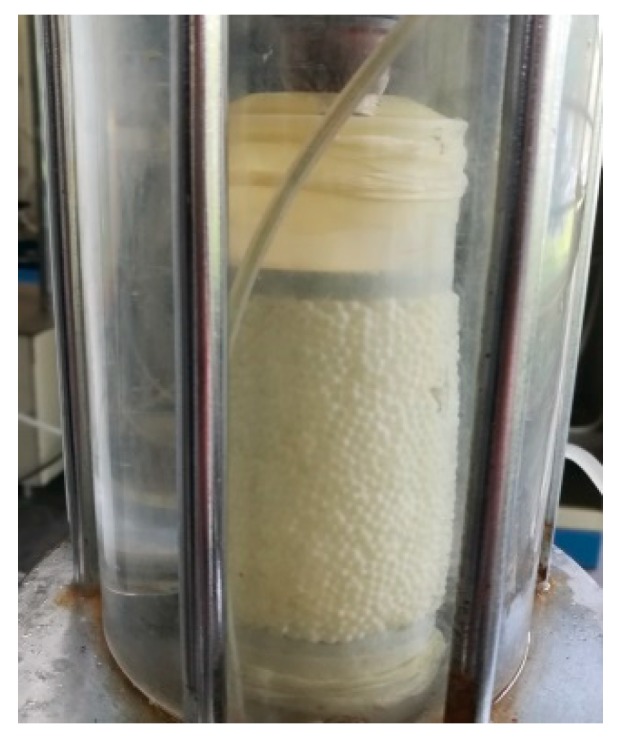
Glass bead specimen in triaxial test.

**Figure 10:**
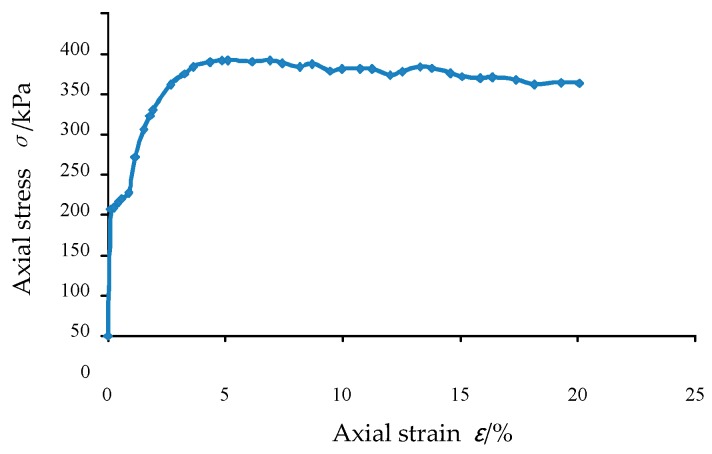
Axial stress-strain curve in triaxial test with 150 kPa confining pressure.

The conventional triaxial test was carried out using glass beads having a particle diameter of 2 mm. The size of the triaxial sample was 3.91 cm in diameter and 8 cm in height and the mass of the particles required for the test was 140 g by the density. The sample was subjected to a confining pressure of 150 kPa, a shear rate of 0.4 mm/min and a sampling step length of 0.2 mm. The sample during the test was as shown in Figure 9. The axial stress-strain diagram obtained during the test was as shown in Figure 10.

**Figure 11:**
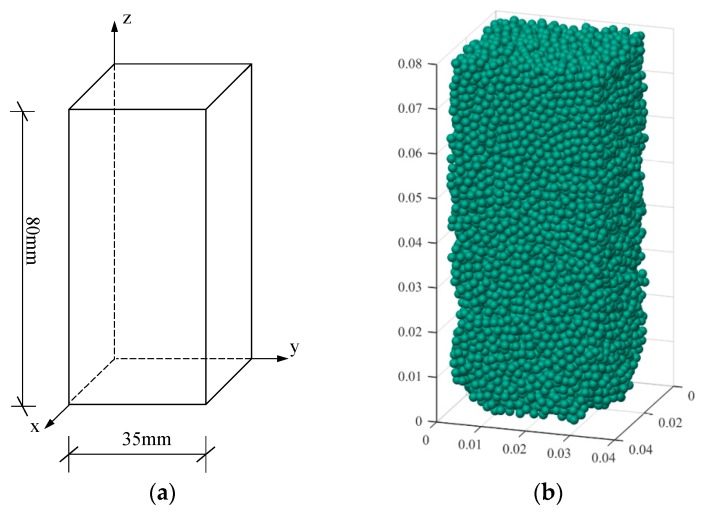
Triaxial test model: (**a**) schematic diagram of triaxial model; (**b**) distribution of particles.

Numerical verification was performed using a triaxial discrete element model with a test size of 1:1. Because the discrete element program is not easy to establish a cylindrical model, the discrete element model is approximated when the discrete element model is established. The size of the established three-axis discrete element model is 35 mm × 35 mm × 80 mm to ensure the cross-sectional area is consistent with the height, as shown in Figure 11. The particle diameter is 2 mm and the irregular arrangement is adopted. The number of particles is 11,636. The parameters used in the numerical simulation are shown in Table 3, Table 4, Table 5 and Table 6.

**Figure 12:**
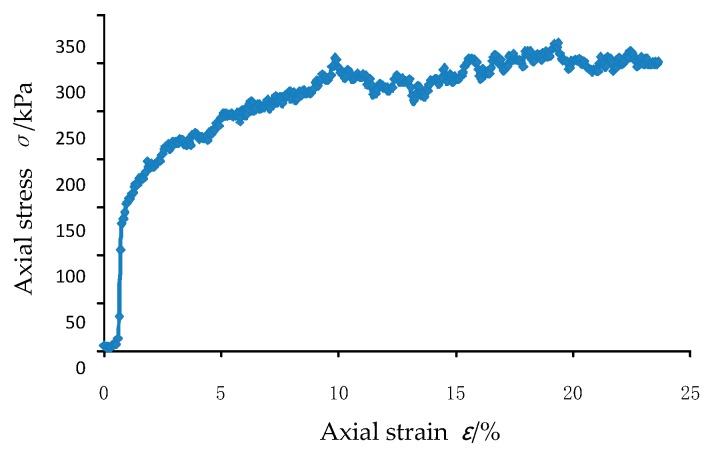
Axial stress-strain curve of the DEM analysis for triaxial test.

During the loading process, the confining pressure is 150 kPa, the upper and lower axial loading rate is 0.01 m/s and the confining pressure application mode adopts the rigid boundary. The stress-strain curve during loading is shown in Figure 12. It can be seen from the axial stress-strain curve that the peak stress is 320.25 kPa.

From the stress-strain curve of the conventional triaxial test, it can be seen that when the axial strain reaches 5.11%, the glass bead sample reaches a peak stress of 342.4 kPa, which is consistent with the peak stress of 320.25 kPa of the three-axis simulation result. Comparing Figure 12 and Figure 10, it can be seen that both trends are first increased and then decreased and the axial stress reduction amplitude is small but the strain corresponding to the peak value in the simulation result is large. According to this, it can be seen that the three-dimensional discrete element simulation effect is good and the initial parameters in the second section are reasonable, which can accurately reflect the force characteristics between the particles.

## 4. Discrete Element Numerical Simulation of Plane Strain Test

### 4.1. The Plane Strain Test

**Figure 13:**
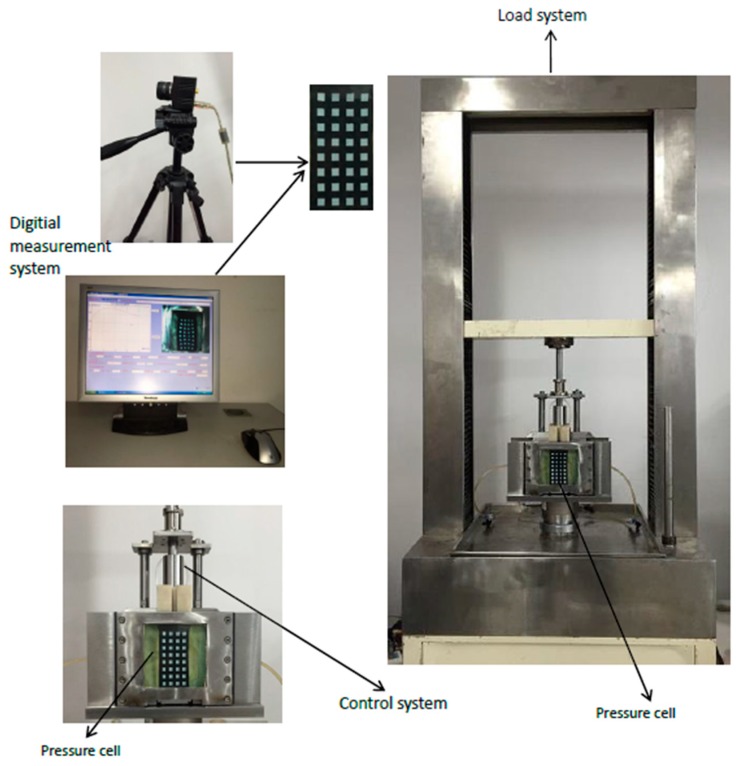
Plane strain apparatus.

A plane strain apparatus is used to study the mechanical properties and shear band failure of soils and has the advantages of flexible loading for lateral confining pressure and noncontact, high accuracy measurements for surface deformation. Figure 13 shows the plane strain apparatus system, the details of which have been described elsewhere [36]. The plane strain gauge based on digital graphics measurement technology is composed of loading system, pressure chamber, control system, digital image measurement system and some other accessories. The loading system includes axial loading system and pressure chamber confining pressure loading system. The digital graphic measurement system is mainly composed of CMOS (Complementary Metal Oxide Semiconductor camera, 5D Mark III, Canon information technology Co. LTD, Beijing, China), image measurement software (V2.0, Dalian University of Technology, Dalian, China) and computer. The sample size of the new plane strain gauge is 100 mm (height) × 60 mm (width) × 100 mm (thickness) and the rubber film side of the sample is a square grid with black and white phase, which is convenient for the acquisition of deformation photography information of the sample and realizes the combination with the digital image measurement system.

**Figure 14:**
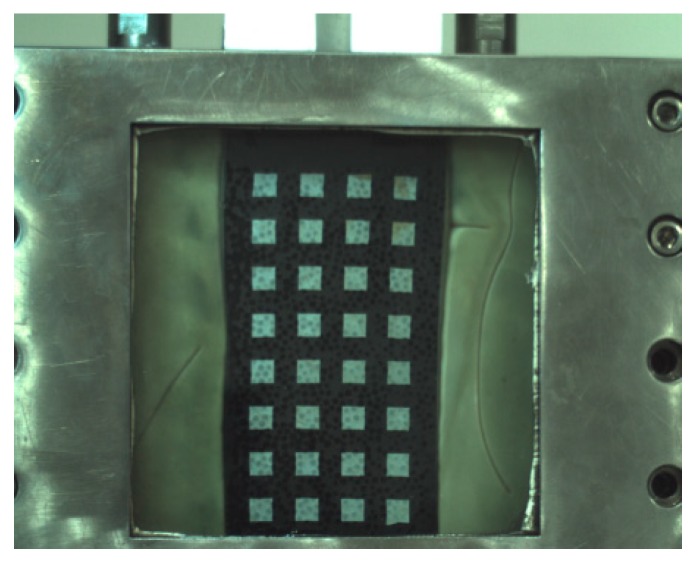
Glass bead sample in plane strain test.

Next, the same glass beads were used for the plane strain test. The glass beads were first cleaned, air-dried, then dried and placed until the glass ball was cooled and the test was started. The sample loading quality can be calculated according to the number of particles, glass bead density and glass bead particle size in the simulation process. Considering that the weight of glass bead and the arrangement rules of particles are difficult to be guaranteed in the process of sample loading, the quality of the sample selected is slightly higher than that required in the simulation, which is 926 g. The confining pressure and simulated confining pressure used in the test were consistent at 150 kPa and the shear rate was controlled at 0.1 mm/min = 0.006 m/h during the test. The image acquisition rate was 12 sheets/min. The glass bead sample of the plane strain apparatus pressure chamber during the test is shown in Figure 14.

**Figure 15:**
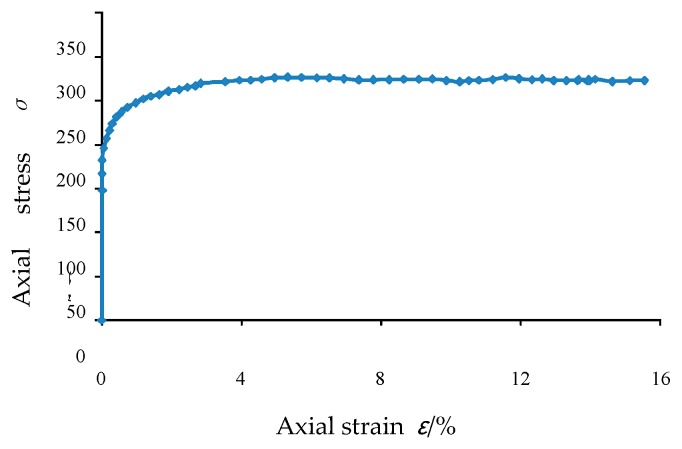
Axial stress-strain curve in plane strain test with 150 kPa confining pressure.

The axial stress-strain diagram obtained during the test is shown in Figure 15. It can be seen that when the axial strain is 5.32%, the axial stress reaches the maximum value of 276.83 kPa, while the later axial stress gradually decreases steadily.

### 4.2. Discrete Element Numerical Simulation for Plane Strain Test

**Figure 16:**
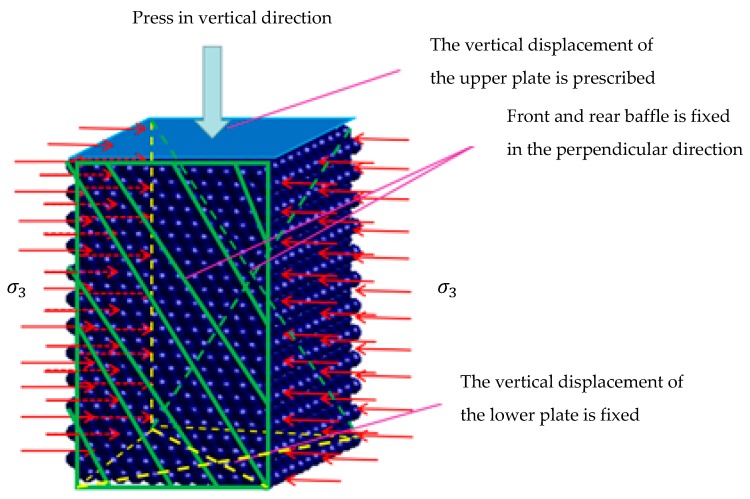
The discrete element numerical model for plane strain test.

A plane strain discrete element particle model with a size ratio of 10:6:10 is generated. On the top and bottom of the model, two virtual thin plates parallel to the particle surface are used to exert axial displacement loads on the model. Fixed boundaries are applied on the front and back sides to limit the displacement of particles in the front and back directions. The confining pressure is applied to the plate on both sides of the sample and then transmitted. The confining pressure is applied directly to the boundary particles. In this way, the specimen can be consistent with the plane strain test in the process of compression. The discrete element numerical model is shown in Figure 16.

The time step formula used in this discrete element model

(11) $$dt=\frac{\sqrt{\rho \left(\pi^{2}+{\left(\mathrm{lg}COR\right)}^{2}\right)}}{\sqrt{3E}}\times 2r_{\min}$$

where *ρ* is the density of the particle; *COR* is the restitution coefficient between particles; *E* is Young’s modulus of particles; *r*_min_ is minimum radius of particles.

Formula (11) shows that the time step is proportional to the particle size, that is, the total calculation time is inversely proportional to the particle size. Studying the effect of size effect in plane strain model is helpful to improve the efficiency of calculation in the process of later calculation and simulation. That is to say, it is not necessary to carry out numerical simulation according to the actual particle size but to calculate the results of small particle size simulation according to the numerical simulation results of large particle size and the rule of particle size effect.

In the numerical simulation of plane strain, numerical simulation has been affected by particle size due to factors such as computer memory and computer calculation efficiency. Generally, the smaller the particle size, the longer the calculation time. Studying the effect of the size effect in the plane strain model is beneficial to improve the calculation efficiency in the subsequent simulation process. That is, when performing numerical test comparison, it is not necessary to perform numerical simulation in full accordance with the actual particle size. Rather, the model and particle size are expanded by equal magnification and the small particle size simulation results are estimated by numerical simulation results of large particle size and the influence of particle size on numerical simulation.

The number of model particles used in the discrete element numerical simulation of the plane strain test is approximately equal to the number of test particles. For the plane strain test of the glass sphere, the number of glass beads can be basically determined. The discrete element simulation time is inversely proportional to the radius of the granular material, which means that the smaller the radius of the particle material, the longer the numerical simulation time. In this regard, the proportional expansion of the radius of the particle material and the size of the model are taken to increase the computational efficiency. As the size of the plane strain test sample is 10 cm × 6 cm × 10 cm, the sample material is glass bead with a diameter of 2 mm, so the discrete element model size we adopted is 50 m× 30 m × 50 m, 100 m × 60 m × 100 m, 200 m× 120 m × 200 m, 400 m× 240 m × 400 m, 500 m × 300 m × 500 m, 600 m × 360 m × 600 m, 700 m × 420 m × 700 m, 800 m × 480 m × 800 m, 900 m × 540 m × 900 m and so forth. The corresponding particle sizes were 1 m, 2 m, 4 m, 8 m, 10 m, 12 m, 14 m, 16 m and 18 m respectively and the non-uniformity of the particles remained the same. The plane strain models of different model sizes were numerically simulated to explore the influence of the model size and particle size ratio on the axial stress-strain relationship of the model when the number of particles is constant. And through the axial stress-strain curve in the simulation process, the influence of the particle size change on the peak stress and the corresponding strain is obtained when the number of particles is constant.

**Figure 17:**
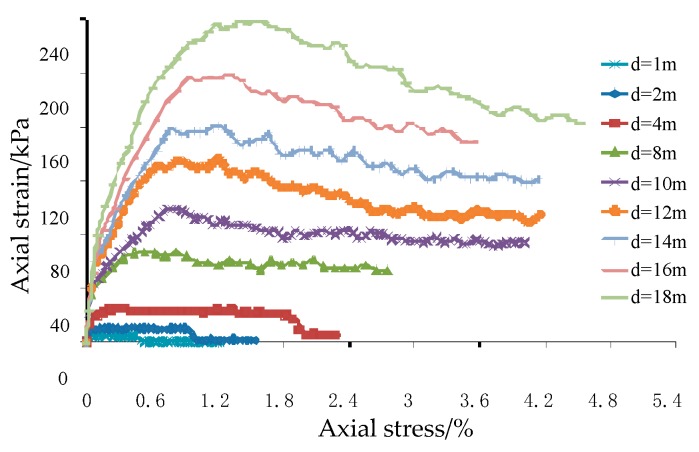
Axial stress-strain curve in plane strain numerical analysis.

**Figure 18:**
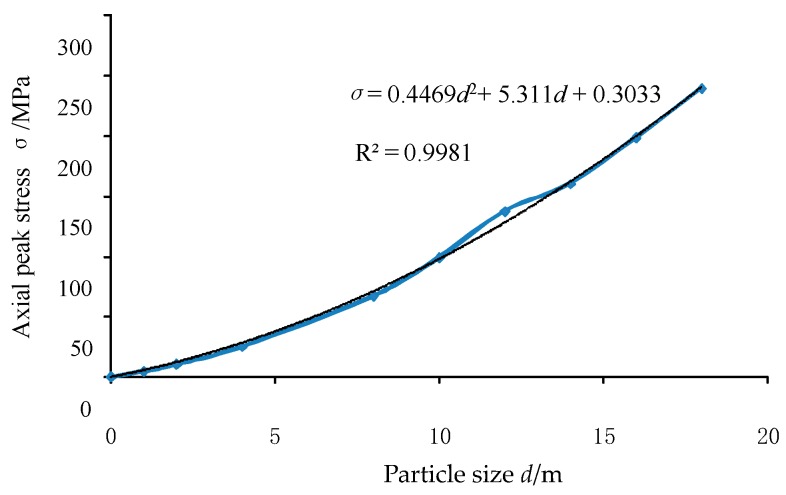
Peak stress-particle size curve in the plane strain numerical analysis.

The axial stress-strain curves in different dimensional plane strain models are shown in Figure 17. It is easy to conclude that the axial stresses first reach the peak and then gradually decrease. As the size of the model increases, the peak stress in the axial stress-strain curve increases gradually and the axial strain corresponding to the peak stress also increases. In the third section, the results of the triaxial simulation are known. The four-prism model particles are used for simulation, which is analogous to the plane strain model. Therefore, when fitting the peak stress *σ*- particle size *d* relationship curve, in order to obtain a better effect at a small particle size, a point (0.002 m, 320.25 kPa) is added. It can be seen from Figure 18 that when the number of particles is constant, the particle size *d* and the model size change simultaneously, the peak stress σ changes quadratically with the change of the particle size *d*, which can be expressed as Equation (12).

*σ* = 0.4469*d*^2^ + 5.311*d* + 0.3033 (12)

According to the curve results fitted in Figure 18, *d* = 0.002 m was substituted into Equation (12) and the corresponding stress *σ* = 0.313 MPa = 313 kPa was obtained. That is to say, according to the fitting results, when the plane strain model size was 10 cm × 6 cm × 10 cm and the particles with 2 mm glass bead parameters were used for numerical simulation, the corresponding axial loading peak stress under the 150 kPa confining pressure was 313 kPa. The corresponding peak stress of the 150 kPa confining pressure obtained by the combined test is 276.83 kPa. It can be concluded that the plane strain simulation results are roughly consistent with the test results.

## 5. Conclusions

This paper developed a slant plate flat throw test system and a sliding friction test instrument for measuring the restitution coefficients and friction coefficients between the glass beads and the boundary surface respectively. The three-dimensional discrete element numerical simulation was performed according to the measured restitution coefficients and friction coefficients. The simulation results are nearly consistent with the experimental results and the feasibility and rationality of the parameters calibration with the presented methods are illustrated.

Compared with other testing methods, the methods in this paper are quite different in experimental objects and methods. The main characteristics are as follows: the use of a flat throwing motion to measure the restitution coefficient, rather than a free fall, ensures the stability of the vertical velocity when in contact with the impact plate; the method for measuring the friction coefficient is simple and feasible and is suitable for testing the friction coefficient between small particles and between particles and boundaries of the devices in laboratory.

As the number of particles is similar to the number of experimental particles in the plane strain numerical simulation and the ratio between the particle size and the model size remains unchanged, the axial peak stress gradually increases with the increase of the particle radius and model size and shows a quadratic curve change with the grain size. From the comparison between the numerical results and the experimental results, the peak stresses of these two are not very different, which shows that the proposed discrete element numerical simulation of the triaxial experiment and the plane strain experiment is reasonable. The measured results can provide parameters for DEM, which can be combined with numerical simulation to verify the feasibility and rationality of the designed measurement methods and the discrete element numerical simulation and emphasize their further application.

As the feasibility study stage of the methodology presented in this study, this paper mainly tests the parameters of the glass bead and simulates the triaxial test and plane strain test in laboratory with proposed DEM. In order to further apply the measuring methods developed in this paper to practical problems, the next work will study the testing and numerical simulation of other fine granular materials such as sand.

## Figures and Tables

**Table 1:** Physical properties of glass beads.

Indexes of Physical Property	Hardness/ (kg/mm^2^)	Refractive Index	Circularity	Precision Error
value	720	1.50	99%	<0.02 mm

**Table 2:** Material thickness.

Materials	Porous Stone	Rubber Membrane	Glass Plate	Filter Paper
Thickness/mm	5.10	0.67	16.18	0.53

**Table 3:** Parameters of restitution coefficient between glass beads and various materials.

Collision Material	Glass Bead	Glass Plate with Rubber Membrane Attached	Porous Stone
Average restitution coefficient	0.926	0.580	0.730

**Table 4:** Friction coefficients between glass beads and different materials.

Coefficient of Friction between Materials	25 mm/min Friction Coefficient	Quasi-Static Coefficient of Friction
rubber membrane	0.2443	0.2116
filter paper	0.2166	0.1791

**Table 5:** The parameters for discrete element method (DEM).

Parameters	Value
Young’s modulus of glass beads *E*	46.2 GPa
Poisson’s ratio of glass beads υ	0.245
Density of glass beads *ρ*	2500 kg/m^3^
Friction coefficient between glass bead particles *μ_s_*	0.16
Restitution coefficient between glass bead particles *COR*	0.926
Friction coefficient between glass bead particles and filter paper *μ_s_*	0.18
Friction coefficient between glass bead particles and rubber membrane *μ_s_*	0.21
Restitution coefficient between glass bead particles and porous stone *COR*	0.58
Restitution coefficient between glass bead particles and rubber membrane *COR*	0.73
*RNS* (Ratio of normal stiffness to tangential stiffness)	0.5
*RZS* (Ratio of normal damping to tangential damping)	0
*ARF* (a non-dimensional parameter related to the rolling friction moment coefficient)	0

**Table 6:** Parameters of 2 mm glass beads.

Parameters	Confining Pressure	Young’s Modulus	Poisson’s Ratio	Density	Restitution Coefficient between Glass Beads	Coefficient of Friction between Glass Beads
value	150 kPa	46.2 GPa	0.245	2500 kg/m^3^	0.94	0.16
